# A Deep Learning Based Approach for Patient Pulmonary CT Image Screening to Predict Coronavirus (SARS-CoV-2) Infection

**DOI:** 10.3390/diagnostics11091735

**Published:** 2021-09-21

**Authors:** Parag Verma, Ankur Dumka, Rajesh Singh, Alaknanda Ashok, Aman Singh, Hani Moaiteq Aljahdali, Seifedine Kadry, Hafiz Tayyab Rauf

**Affiliations:** 1 Chitkara University Institute of Engineering and Technology, Chitkara University, Rajpura 140401, India; parag_verma@yahoo.com; 2 Computer Science & Engineering, Women Institute of Technology (Govt.), Dehradun 248007, India; ankudumka2@gmail.com; 3 SEEE, Lovely Professional University, Jalandhar 144001, India; 4 Computer Science & Engineering, College of Technology, G.B. Pant University of Agriculture and Technology, Pantnagar 263153, India; alakn@rediff.com; 5 School of Computer Science and Engineering, Lovely Professional University, Kapurthala 144411, India; 6 Faculty of Computing and Information Technology, King Abdulaziz University, Jeddah 37848, Saudi Arabia; Hmaljahdali@kau.edu.sa; 7 Faculty of Applied Computing and Technology, Noroff University College, 4608 Kristiansand, Norway; skadry@gmail.com; 8 Faculty of Engineering & Informatics, University of Bradford, Bradford BD7 1DP, UK; h.rauf4@bradford.ac.uk

**Keywords:** COVID-19, machine learning, pneumonia, deep learning model, location attention network, convolution neural network

## Abstract

The novel coronavirus (nCoV-2019) is responsible for the acute respiratory disease in humans known as COVID-19. This infection was found in the Wuhan and Hubei provinces of China in the month of December 2019, after which it spread all over the world. By March, 2020, this epidemic had spread to about 117 countries and its different variants continue to disturb human life all over the world, causing great damage to the economy. Through this paper, we have attempted to identify and predict the novel coronavirus from influenza-A viral cases and healthy patients without infection through applying deep learning technology over patient pulmonary computed tomography (CT) images, as well as by the model that has been evaluated. The CT image data used under this method has been collected from various radiopedia data from online sources with a total of 548 CT images, of which 232 are from 12 patients infected with COVID-19, 186 from 17 patients with influenza A virus, and 130 are from 15 healthy candidates without infection. From the results of examination of the reference data determined from the point of view of CT imaging cases in general, the accuracy of the proposed model is 79.39%. Thus, this deep learning model will help in establishing early screening of COVID-19 patients and thus prove to be an analytically robust method for clinical experts.

## 1. Introduction

In late 2019, a sudden outbreak of pneumonia was detected in Hubei, Wuhan, China, which immediately triggered an unprecedented worldwide outbreak [1,2]. This surprising pneumonia used a deep sequencing probe that discovered evidence of a bat-origin coronavirus known as the novel coronavirus (nCoV-2019) [3,4]. This type of infection is also known as severe acute respiratory syndrome coronavirus 2 (SARS-CoV-2) [2,5]. As of 12 March 2020, the outbreak of this infection has led to approximately 80,981 confirmed cases in various research centers in 31 provinces of China, with approximately 15,741 serious cases, 1972 fatal cases, and 6578 suspected cases [6]. Later, the pandemic covered a wide area across the world including countries such as the Republic of Korea, Japan, Italy, Thailand, France, Iran, Spain, and the United States. As of 31 January 2020, the COVID disease was declared a “Public Health Emergency of International Concern” by the World Health Organization [7]. The process of high transmission of this infection has linked the disease to previous 21st century outbreaks of the beta coronavirus, SARS-CoV [8,9] and the Middle East respiratory syndrome coronavirus (MERS-CoV) [7,10].

This novel coronavirus has shown potential for production during person-to-person transmission [11,12]. Initially, the mortality rate of people affected by this virus was very low compared to SARS-CoV and MERS-CoV, but in recent months its effect has been considered to be very dangerous due to its different variants, in which the death rate of affected people has increased. Social distancing and sanitization have been found to be the best way to avoid this virus so far. Many studies have been conducted by various research centers and clinical trials around the world to achieve 100% eradication of this virus, but none of the research has reached that benchmark. In view of the way this epidemic is continuously changing its behavior, immense possibilities of research are being seen in this pandemic. Prodromal dry cough and low body temperature are one of the main features of SARS-CoV-2 infection [13]. Because symptoms similar to this virus are also found in influenza A and influenza B viruses, the clinical diagnosis of COVID-19 pneumonia can be difficult. For high volume detection of suspected COVID-19 cases, laboratory detection is challenging and may not be accessible to everyone with the associated contamination and those who can also be infected, due to the lack of a test package for SARS-CoV-2.

CT images play an important role in the analysis of COVID-19 pneumonia. CT images are also considered as primary line imaging methods in deeply determined cases and are useful for visualizing image changes during treatment. Therefore, it can be concluded that CT images can be considered as an effective diagnostic analytical tool for persons suspected of having COVID-19 with negative reactions of reverse transcription-polymerase chain reaction (RT-PCR), and that they have the ability to identify individuals who are exceptionally suspected of SARS-CoV-2 infection [6]. Findings on CT images may indicate disease severity. Therefore, artificial intelligence based prognostic techniques may be helpful in identifying COVID-19 pneumonia.

Deep learning techniques have demonstrated usefulness in fields that depend on image-based data, e.g., radiology, pathology, dermatology and ophthalmology [14]. Instead of regular subjective visual evaluation of images by trained practitioners, deep learning optimization separates complex examples into resulting information and subsequently provides assessment in a quantitative way. Conversely, when feature engineering is approached, deep learning systems take automatic qualification and selection of features, and thus hardly expect human interaction. Deep learning techniques have identified their feature engineering counterpart’s in several tasks, including identifying mammographic lesions [15], the prognosis for mortality [16] and multi-modal imaging enlistment.

Convolutional neural network (CNN) modeling is a class of deep learning techniques that link imaging filters to simulated channels with artificial neural networks (ANNs) through a series of progressive linear and non-linear layers. The CNN layers progressively capture the most prominent levels of images, eventually mapping the approximate decision-making of the images to the original desired output. CNN has shown exceptional handling ability of photographic, pathological and radiographic images in order to classification [17], detection [18], segmentation [19], registration [20] and reconstruction [21] as well. Pre-trained associations are used in images from different regions with different approaches; these approaches are known as transfer learning, as an alternative solution when the test size is considered low. In some instances, classifiers are constructed using an ensemble of deep learning and feature engineering.

**Figure 1:**
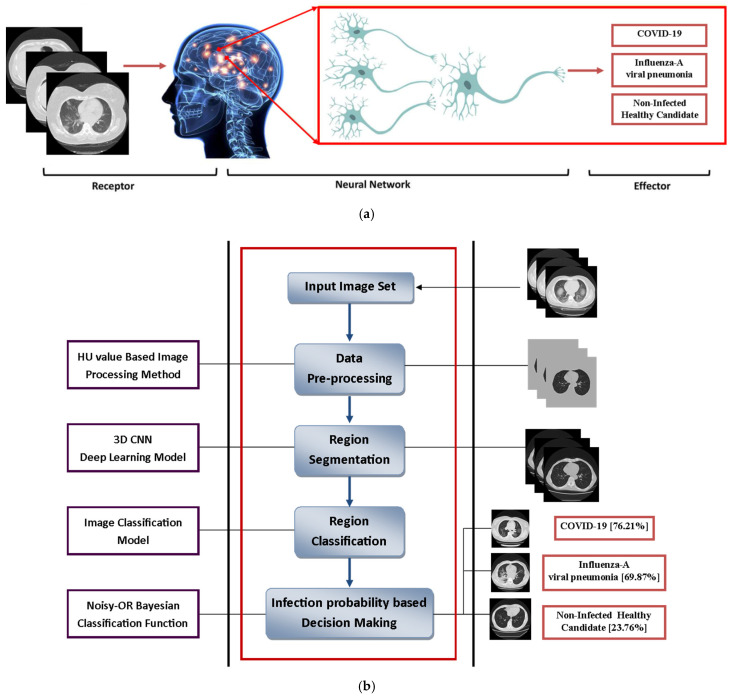

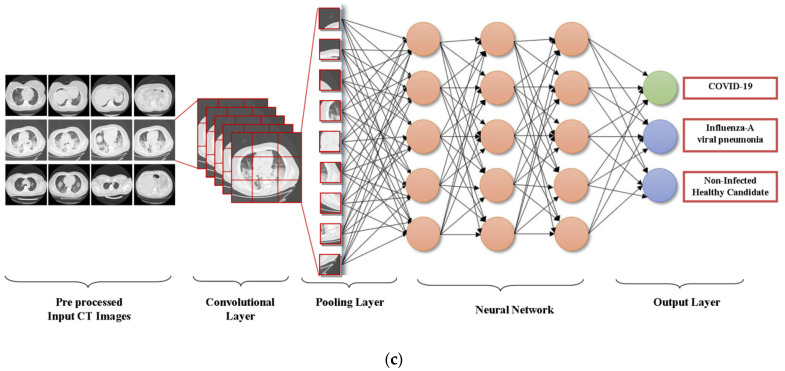
(**a**) Concerned neural network with human deep learning module, (**b**) modeling of research structure, (**c**) a convolutional neural network (CNN) model.

The aim of this research is to explore the potential of deep learning networks, especially 3D CNNs, to measure chest CT features as claimed by the research center with the detection of COVID-19 features. This research can help in the evaluation of suspected cases and claimed cases of COVID-19. This structured research has a broad scientific agreement (Figure 1), in which 7 small and specific CT-based COVID-19 cases were analyzed. To detect and validate the prognostic intensity of CNN’s model, it considers three classes including COVID-19, influenza A viral pneumonia, and non-reflective healthy cases. We compared the performance of the CNN model with models based on clinical parameters and engineered features, as demonstrated by its robustness in test trials and user-variable conditions. To enhance the understanding of the assets captured by the CNN model, we map notable regions in the images according to their commitments to predict within and outside COVID-19.

## 2. Related Works

Given the previous research work relating to statistics on 2019-nCoV data, there are very few. This area, therefore, deserves the attention of researchers to contribute to the better prediction of 2019-nCoV data. Researcher Beck et al. [22] proposed architecture for prediction of drug-target interactions (DTIs) based on the concept of artificial intelligence (AI) which is useful for controlling the rapid spread of these infectious diseases. In designing the architecture, molecule transformer-drug target interaction (MT-DTI) has been used to estimate the binding affinity value between commercially available antiviral drugs and target proteins, in another study researcher Nesteruk [23] using a mathematical model to estimate the characteristics of 2019-nCoV in China.

The authors used a time versus individual susceptible, infected, and removed (SIR) model for objective prediction. The obtained results of the correlation coefficient are highly appreciated, but because of some weak features of the method that estimate the suspect requires additional analysis before making a decision. The researchers Majumder and Mandl [24] proposed a model called incidence decay and exponential adjustment (IDEA) of mathematics, which uses average serial interval lengths ranging from 6 to 10 days from SARS and MERS to fit the IDEA model. Researcher Read et al. [25], proposed a mathematical transmission model assuming a latent period of four days and close to an incubation period. For statistical calculations, this model uses venom distribution and then applies a deterministic SEIR meta-population transmission model over daily time increments with transmission rate =1.94 and infectious duration =1.61 days. Similarly in one other study researcher Butt et al. [26] proposed a deep learning-based complementary diagnostic method for frontline clinical doctors with 86.7% accuracy to classify symptoms into three groups, including COVID-19, influenza A viral pneumonia, or healthy infected patients.

## 3. Materials and Methods

### 3.1. Source of Dataset and Description

For modeling purposes, we extracted the computed tomography images from the publicly accessible COVID-19 cases data, which is accessible by radiopedia.

The research took into account a total of 548 cross-segment CT image samples that have been collected online. Out of these 548 CT images, 232 images are of 12 patients infected with SARS-CoV-2 infection, 186 CT images of 17 patients infected with influenza A virus, while the remaining 130 CT images are of 15 patients found in the category of non-infected healthy candidates. All the COVID-19 infected patients were identified and confirmed through a real-time polymerase chain reaction (RT-PCR) test package. During this investigation, only those cases whose chest can be seen in the CT image are included. The median age of patients with confirmed infection was 46 years (which ranged from 18–69 years), and of whom approximately 73% were male. Symptoms such as mild fever, conjunctivitis, dry cough, skin rash, and fatigue were observed in most of the patients, while 23% of the patients had symptoms of severe illness such as chest pain or pressure, and difficulty in normal breathing.

**Figure 2:**
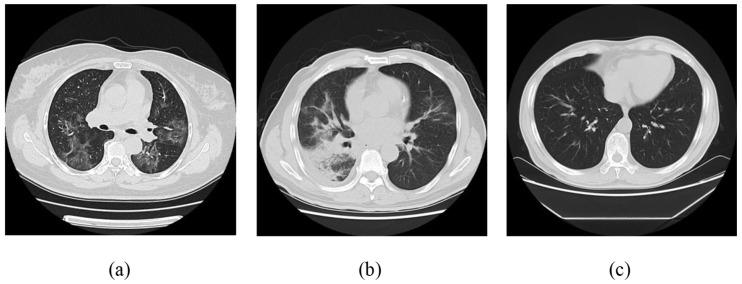
Different sections of the transverse on CT image; (**a**) on the novel coronavirus; (**b**) on influenza A viral pneumonia (IAVP); (**c**) on a healthy candidate who is not infected with the virus.

According to this study, it suggests that there should be a gap of at least one day between CT image data sets taken from the same patient to ensure different types of growth of virus. Thus the 316 CT images under research were obtained from the First Affiliated Hospital of Zhejiang University as a controlled analysis collection. Out of which 186 were collected from 17 patients infected with influenza A virus who have CT images, including A(H1N1), A(H3N2), A(H5N1), A(H7N9), etc., and 130 CT test images were of healthy candidates who were found non-infected during the test. The study revealed that 232 (87.62%) and 186 (81.69%) cases of COVID-19 and influenza A respectively were reported from the early or progressive stage, while the remaining 9.6% and 13.4% were from the severe stage (p>0.05). Additionally, cases of influenza A virus are being isolated from cases of patients suspected of severe SARS-CoV-2 infection (e.g., cases considered in Figure 2a,b currently worldwide).

### 3.2. Dataset Preprocessing

To promote COVID-19 recognition, the CT image was returned to keep the tone of the CT image at 1×1×1 mm^3^ that followed the nearest neighbor insertion standard. At the time, CT sets that were returned to create viable lung region cover were pre-processed to place random locations before preparation for the deep learning model.

**Figure 3:**
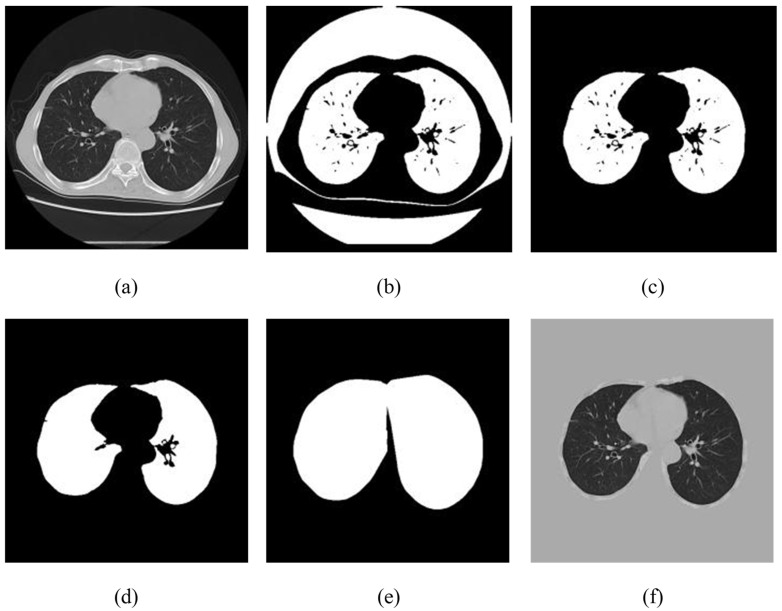
Resulting images of the preprocessing steps: (**a**) re-sampling of CT images with normalization; (**b**) CT image binarization with HU threshold ~600 estimated; (**c**) after the separation of unrelated areas; (**d**) after the application of erosion and dispersion operation; (**e**) mask construction on the image via convex hull operation; (**f**) fusing image of (**a**) and mask (**e**) to generate a valid pulmonary region.

1. As the advanced grayscale image was ranging in pixel estimates (0, 255), the retuned raw data of the CT image was appropriately transformed from the Hounsfield unit (HU) to the aforementioned properties. The HU data frame was truncated to within (−1200, 600) (any value other than this was set to −1200 or 600) and then standardized to (0, 255), directly corresponding to the digital format as presented in Figure 3a.
2. A fixed range (−600) was used to remove or binarize the returned computed tomography images, and delicate bones and tissues, e.g., nerves and muscles, were filtered with considerable HU values Figure 3b.
3. Each associated segment smaller than 0.3 cm^2^ and more than 0.99 unpredictable was ejected to remove some noise from high-brightness spiral imaging. The segment (usually clothes and ancillary equipment other than the human body) was also evacuated with center point separation of the CT image over 7 cm. In addition, segments with volumes of 450 and 7500 cm^3^ were retained, as seen in Figure 3c. In the current investigation the range was increased to the contrary and those who announced the discovery of the lung knob were 22, which ranged from 680 to 75% cm^3^. Knob detection study typically focuses on small spots, while viral infection can be progressively for COVID-19 cases.
4. In step 3, the mask disintegrated into two separate regions and then expanded to the original shape to remove the small black spots Figure 3d.
5. High-structure activity, i.e., convex hull operation was performed on the effective region to include areas of viral disease associated with the external mass of the lung, which had been removed from previous advances.
6. Image matrix data from step 1 was replicated by the exchanged masks from step 5 to obtain the final powerful aspiration region for further preparation. The region outside the mask was filled with 193, which was proportional to 0 when it returned to the HU estimate as Figure 3f.

### 3.3. Data Processing and Augmentation Process Segment

Big amounts of the uninfected region were also isolated for this examination using a 3D division model, including the fibrotic structure of incorrectly recognized pneumonic, calcification, or non-infected regions. Thus, an additional class was added for disease as a non-infected region (NIR) apart from influenza A virus and COVID-19.

So, under this finding, an additional classification was added to group the disease as n-infectious region (NIR) is added despite COVID-19 and influenza A viral pneumonia. The examination included total 618 CT test images (having contribution of 278 cases of COVID-19, 224 cases of influenza A viral pneumonia and 175 cases of NIR). Therefore, 3957 applicative 3D shapes were created from the 3D segmentation model; only this 3D square structure had more outrageous information on the middle mark of progress closer to the center. In addition, the central image with two neighbors of every 3D shape found this district to address for a possible classification step. Then, at that time, all the patches of the image were arranged by two specialist radiologists into two types: non-infectious disease and pneumonia. The images were commonly viewed as COVID-19 or influenza A viral pneumonia, depending on the results of clinical findings in the final classification.

Cumulatively, a total 548 patches of image were obtained and considered for final experiment from the above propels, that includes 232 for COVID-19, 186 for influenza A viral pneumonia and 130 for NIR or healthy candidates, which were found to be non-infected. Based on the previous data indices, the data has been divided into 70% as training and 30% as test dataset for model; individually, a total of 383 CT sample tests have been included under training dataset, including 165 of COVID-19, 131 of influenza A viral pneumonia, and 87 for healthy candidates. An additional 165 (30%) images were kept somewhat different to the test dataset.

Currently, the possibility of testing for cases of COVID-19 and influenza A viral pneumonia has been doubled to accommodate the number of simulated infection instances to reduce the impact of disproportionate spread of the different types of images in the data set. Additionally, non-specific information expansion tools, for example, random clipping, flipping (left–right, and up–down), and reflection operations, extend the amount of test preparation in samples and prevent information from being adjusted.

### 3.4. The deep Learning Model for Classification and Segmentation

#### 3.4.1. Region-Based Classification Technique

**Figure 4:**
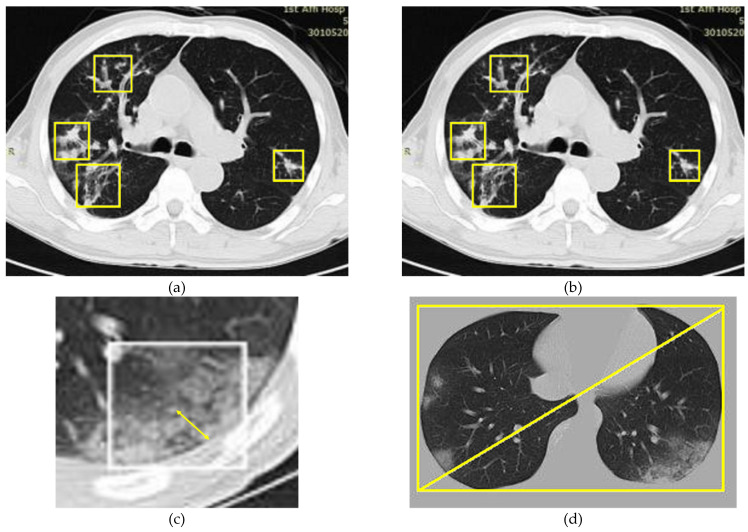
(**a**) Four ground-glass focus of infection on the novel coronavirus image; (**b**) similarly, the ground-glass image of influenza A viral pneumonia focused on infection; (**c**) identification of the distance (minimum) between the mask and the center of the patch (presented with a two-headed arrow); (**d**) diagonal identification of the bounded rectangle (with the minimum length) of the pulmonary image.

A designed purposed by researcher Kanne [27] and Chung [28], considered three distinct features of COVID-19: the presence of pleura-glass, marginal diffusion next to the pleura, and there is usually more than one transition free focal region, the case as shown in Figure 4.

Our model based on optimizing these results. The purpose of the image classification model is to identify the presence and structure of transformed diseases. In addition, the relatively sharp edge shape was used as an additional weight to familiarize the model with the concern area data relative to the correction on the pneumonic image. The focal area of infection found near the pleura was more likely to be considered as COVID-19.

Relatively sharp edge shapes of each arrangement were determined as follows:

- Calculate the shortest separation from the mask for the middle of this classification (the double head arrow displayed in Figure 4c).
- A rectangular shape with minimum diagonal length is obtained from the target image (as shown in Figure 4d).
- Then, the normal area from the covered edge at that point is obtained from the distance estimated from step 1 numerically divided by the inclination of step 2).

#### 3.4.2. Applied Network Structure

**Figure 5:**
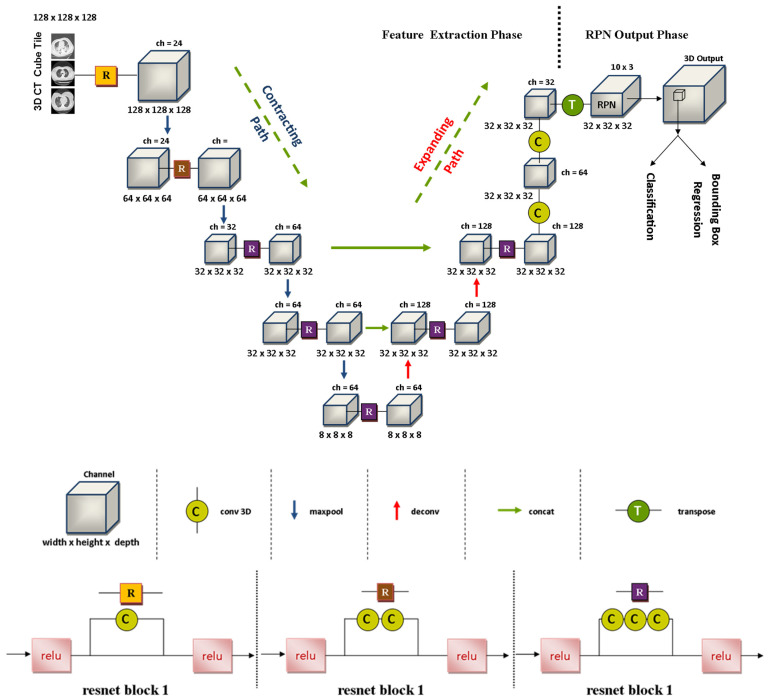
Block Structure of the VNET-RPN Network.

As per the widely used of 3D V-NET (Volumetric-Convolutional Network) in the field of medical imaging segmentation, we are going to use the VNET-RPN (Volumetric-Convolutional network) structure (as of Figure 5) in current work.

It involves two forms of the system: contract and extension paths. In the first step images are fed into the contracting path to complete the down examination process for capturing configuration data.

At that point, the precision-image up examination process completes in a balanced and extended way to obtain accurate image constraint data. Simultaneously, the features map with equivalent measurements of both paths are interconnected, which encourages maintaining the point-by-point of the neural network system of the contracting pathway during the production of information.

The output layer of 3D V-NET must be suppressed, as they were originally intended to create image segmentation, while the focus of this research was to locate and classify the region of viral infection. This implemented evaluation model consisted of two sections: including the feature extraction section and the region proposal network (RPN) performance layer. The first section extracted the features, i.e., generated feather maps and allow the network to capture data at multiple scales. In the next section RPN’s performance settings allowed the system to offer resolutions (jump boxes for predicted regions) and classification directly.

Through research, we structured and evaluated a 3D CNN system model, which has a multi-feature extraction structure with a comparative RPN layer, with a V-NET backbone as part of feature extraction, as it appears in Figure 5.

### 3.5. Analytical Report of Network

#### 3.5.1. The Decision for Each Candidate Region

This artificial intelligence technique is inspired by the hypothesis of bagging production algorithm based on the ensemble technique of machine learning. In it, a candidate region is addressed through three patch images: origin image consist of two neighbors. These three images decide the innovation with this whole region.

1. In the event that in any condition if two pictures were classified into a comparative type, the image with the most extreme confidence level at that time was chosen.
2. Otherwise, image with maximum confidence level (not overwhelmed) was chosen.

In the next step, the region with a non-infected region for disease was neglected.

#### 3.5.2. Noise-OR Bayesian Classifier Function (Cause of Normal Reports)

One of the unique features of the spreading nature of COVID-19 is more than one independent focal area of infection involved in the case of CT image test. It is sensible that if a patient has two COVID-19 regions, the overall probability is more than half, meaning both have being half-chances. As needed, the absolute transition confidence level (P) for disease type was determined using a Noise-OR Bayesian classifier of probability (Equation (1)):

(1) $$P=1-\underset{i}{\prod }\left(1-P_{i}\right)\;$$

where, $P_{i}$ presents the confidence level of the *i*th region.

In the same way, two types of P COVID-19 and P influenza A viral pneumonia, were concluded, at this point, this CT test was categorized in the comparison group as indicated by the estimated value of P.

In addition, procedures that were used for a sensible referral to clinical experts with the reliability certainty of a complete CT test were:

1. In the event that both estimates of P were equal to 0, at this point there was a spot in this CT test belongs to the non-infection founded case.
2. If one of the P estimates was equal to 0, then the second P estimate was directly changed to the certainty of this CT test.

In addition, the softmax function was used to create two confidence levels.

(2) $$S_{i}=\frac{e^{P_{i}}}{\sum^{\;}j^{e^{P_{i}}}}\;$$

Softmax function operation standardized the sum of $S_{i}$.

## 4. Results

### 4.1. Evaluation Phase

An Intel Core i7-eighth generation CPU with a practical 4 GB NVIDIA realistic graphic card detail framework has been used to test the proposed model. The processing time is deeply dependent on the number of image layers in a single CT image set. Normally it is taken from preprocessing of information to report output under 30 for CT image set of 70 layers.

### 4.2. Model Training Process

**Figure 6:**
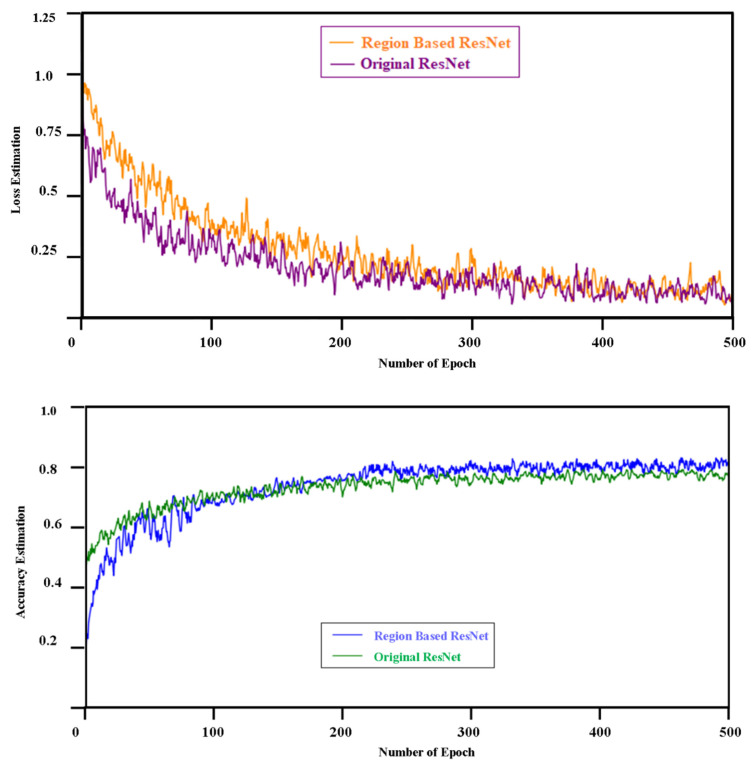
The training curve for the two classification models for estimation of loss and accuracy.

Cross entropy was just used, as one of the more traditional lose function used in the classification model. When the epoch number of model generation training cycles exceeded 500, the estimation of loss value was markedly decreased or increased, proposing that the model may well be in a somewhat idealized state without a particular over-fit value. Modeling training curves to the estimation of loss and accuracy for the two classification models as in Figure 6. The framework contrast preparation of data collection with the region-based system obtained an improved execution in the original ResNet.

### 4.3. Model Performance of Test Dataset

The precision of the model decides how exact the qualities are assessed. The accuracy decides the reproducibility of the gauge or the numbers of expectations are right. The survey shows the number of right outcomes has been found. The f1-score utilizes a combination of accuracy and adjustment to decide unbiased outcomes with average effect. The equations from (3) to (6) show the most ideal approach to compute the performance metrics of the model,

(3) $$\begin{array}{c} \mathrm{Accuracy}\;\left(\mathrm{acc}.\right)\\ =\frac{\left(\mathrm{True}\;\mathrm{Positive}+\mathrm{True}\;\mathrm{Negative}\right)}{\left(\mathrm{True}\;\mathrm{Positive}+\mathrm{False}\;\mathrm{Positive}\right)\left(\mathrm{True}\;\mathrm{Negative}+\mathrm{False}\;\mathrm{Negative}\right)}\; \end{array}$$

(4) $$\mathrm{Precision}\;\left(\mathrm{prec}.\right)=\frac{\mathrm{True}\;\mathrm{Positive}}{\left(\mathrm{True}\;\mathrm{Positive}+\mathrm{False}\;\mathrm{Positive}\right)}\;$$

(5) $$\mathrm{Recall}\;\left(\mathrm{rec}.\right)=\frac{\mathrm{True}\;\mathrm{Positive}}{\left(\mathrm{True}\;\mathrm{Positive}+\mathrm{False}\;\mathrm{Negative}\right)}\;$$

(6) $$f_{1}-\mathrm{score}=\frac{2\times \mathrm{prec}.\times \mathrm{rec}.}{\left(\mathrm{prec}.+\;\mathrm{rec}.\right)}\;$$

### 4.4. Segmentation

**Figure 7:**
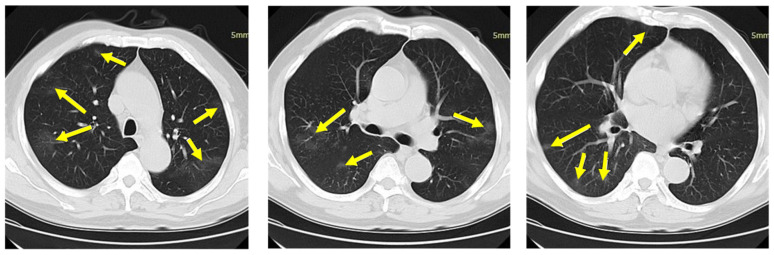
All CT images of a single CT case are shown here. The center of infection in the image is indicated by arrows.

To test the model, a total of 20 CT sample images were arbitrarily selected from each group of CT images, for example COVID-19, influenza A viral pneumonia, and healthy candidate without infection. Following the standard that considered a person image (not the owner of this CT) was included during the model training phase. In addition, the VNET-IR-RPN segmentation model was coordinated to reduce the range of motion, so it can be executed very well even in an increasing number of free regions in many specialized areas. An example of a CT test in which a location was not divided, for example, COVID-19 or influenza A viral pneumonia, was incorrectly classified into a healthy candidate region group, as shown in Figure 7. The contamination is hardly observable by an individual and appears suspicious even for this evaluation to be considered by the model.

### 4.5. Classification for a Single Image Patch

Total 165 patches of different images were obtained from 44 CT sample tests, which includes 69 images for COVID-19, 54 images for influenza A viral pneumonia and 42 images for healthy candidates having no-infection. The design of each interaction was evaluated using the actual and predicted values of confusion matrix. With including and disinclining the region-specific features structures of two models have been evaluated and their corresponding confusion matrix computed in Table 1 and Table 2.

The average f1-score for both models was calculated to be 0.6393 and 0.6633, respectively. Comparing the f1-scores of the models shows that the second models with better performance which include a region specific feature model with the other model are not included. In this sense, this model was used for the remainder of this research exploration.

Rulings for a specific region each image fix chooses to introduce the entire candidate locale regions. An aggregate of 165 candidate cubic structures were perceived, including 69 of COVID-19, 54 of influenza A viral pneumonia, and 42 of healthy candidates without infection. A confusion matrix of the decision results and performance metrics associated accuracy, precision, recall, and f1-score showed up in Table 3 and Table 4. The average f1-score for the three order classification was 0.65 and contrasted with a 4.68% improvement from the past advance.

### 4.6. Classification Results for CT Image Samples

The noise-or Bayesian classifier task was used to separate the predominant disease types. In a previous report, three types of results were analyzed over three categories such as COVID-19, influenza A viral pneumonia, and non-infected regions (NIRs). The results obtained during test are summarized in Table 5 and Table 6. Since the NIR for disease would be ignored and not checked by Bayesian classification function, we only took an average f1-score for the two onsets. They were 0.65 and 0.69 individually, indicating a growth of 6.16%. Furthermore, the general ordering accuracy for each of the three groups is 79.39%.

This research used the deep learning technique in order to identify and classify COVID-19 from influenza A viral pneumonia. With respect to the structure of the system, traditional V-NET was used to feature extraction. Then it tested with system models with and without additional components of region specified features. Testing indicated that the component described above could detect COVID-19 cases more effectively.

## 5. Conclusions

At present there are no excellent diagnostic kits for rapidly identifying COVID-19 and professional doctors are also highly confused in differentiating between normal flu and COVID-19, the unwanted virus that has created fear in every human without geographical limitation. Currently, RT-PCR is the only exclusive option for identifying COVID-19. Here, we utilize the deep learning technique that can be utilized for masses and could help to control the pandemic by identifying COVID-19 patients in order to quarantine them as needed. Our research presents a deep learning technique that could screen COVID-19 automatically. The model uses multicenter case studies for collecting the CT images as the dataset. The region-specific feature mechanism promotes the model that can perform more accurately to classify COVID-19 from chest radiography. The model overall accuracy rate was found to be around 79.39% and to be promisingly advantageous with small sample sizes due to the dearth of data indication techniques for leading clinical experts. However, with an additional sample size in the future, the more accurate results may be produced that will be helpful for researchers and the scientific community in predicting the cases as well as in fighting back COVID-19 itself. In addition, this outcome will also help to identify the other diseases post-SARS-CoV-2 infection and future public health emergency.

## Figures and Tables

**Table 1:** Computation of a 3 × 3 confusion matrix on three attributes as COVID-19, IAVP and a healthy candidate without infection (HCI). The features specified in the presented areas of the V-NET and VNET-RPN models are represented through V_1_ and V_2_.

	Predicted Results					
	COVID-19		IAVP		HCI	
	$V_{1}$	$V_{2}$	$V_{1}$	$V_{2}$	$V_{1}$	$V_{2}$
**COVID-19**	180	174	17	14	40	41
**IAVP**	33	31	193	187	49	53
**HCI**	54	51	45	42	587	608

**Table 2:** Model performance evaluation metrics on recall, precision, and f1-score of both classification models for COVID-19, IAVP, and a Healthy Candidate without infection (HCI).

	Recall		Precision		f1−Score	
	$V_{1}$	$V_{2}$	$V_{1}$	$V_{2}$	$V_{1}$	$V_{2}$
**COVID-19**	0.65	0.64	0.58	0.58	0.59	0.61
**IAVP**	0.64	0.63	0.54	0.56	0.58	0.59
**HCI**	0.73	0.73	0.76	0.75	0.74	0.78

**Table 3:** Confusion matrix of COVID-19, IAVP, and a Healthy Candidate without infection (HCI).

	Predicted Results		
	COVID-19	IAVP	HCI
**COVID-19**	42	16	7
**IAVP**	13	37	8
**HCI**	9	4	29

**Table 4:** Model performance evaluation metrics on recall, precision, and f1-score of both classification models for COVID-19, IAVP, and a Healthy Candidate without infection (HCI).

	Predicted Results		
	Recall	Precision	f1−Score
**COVID-19**	0.66	0.65	0.65
**IAVP**	0.66	0.64	0.64
**HCI**	0.66	0.69	0.67

**Table 5:** Confusion matrix of COVID-19, IAVP, and HCI.

	Predicted Results		
	COVID-19	IAVP	HCI
**COVID-19**	56	8	5
**IAVP**	8	41	6
**HCI**	4	3	34

**Table 6:** Model performance evaluation metrics on recall, precision, and f1-score of Bayesian classification models for COVID-19, IAVP, and a healthy candidate without infection (HCI).

	Predicted Results		
	Recall	Precision	f1−Score
**COVID-19**	0.82	0.81	0.82
**IAVP**	0.79	0.75	0.77
**HCI**	0.76	0.83	0.79

## Data Availability

Not applicable.
